# Fourier-Transform Infrared (FTIR) Spectroscopy for Typing of Clinical *Enterobacter cloacae* Complex Isolates

**DOI:** 10.3389/fmicb.2019.02582

**Published:** 2019-11-06

**Authors:** Sophia Vogt, Kim Löffler, Ariane G. Dinkelacker, Baris Bader, Ingo B. Autenrieth, Silke Peter, Jan Liese

**Affiliations:** ^1^Institute of Medical Microbiology and Hygiene, University Hospital Tübingen, Tübingen, Germany; ^2^German Center for Infection Research (DZIF), Partner Site Tübingen, Tübingen, Germany

**Keywords:** *Enterobacter cloacae* complex, Fourier-transform infrared spectroscopy, bacterial typing, whole genome sequencing, artificial neural network, outbreak

## Abstract

Members of the *Enterobacter* (*E.*) *cloacae* complex have emerged as important pathogens frequently encountered in nosocomial infections. Several outbreaks with *E. cloacae* complex have been reported in recent years, especially in neonatal units. Fast and reliable strain typing methods are crucial for real-time surveillance and outbreak analysis to detect pathogen reservoirs and transmission routes. The aim of this study was to evaluate the performance of Fourier-transform infrared (FTIR) spectroscopy as a fast method for typing of clinical *E. cloacae* complex isolates, when whole genome sequencing (WGS) analysis was used as reference. First, the technique was used retrospectively on 24 first isolates of *E. cloacae* complex strains from neonatal patients and showed good concordance with SNP-based clustering [adjusted rand index (ARI) = 0.818] and with the sequence type (ST) (ARI = 0.801). 29 consecutive isolates from the same patients were shown by WGS analysis to almost always belong to the same SNP cluster as the first isolates, which was only inconsistently recognized by FTIR spectroscopy. Training of an artificial neural network (ANN) with all FTIR spectra from sequenced strains markedly improved the recognition of related and unrelated isolate spectra. In a second step, FTIR spectroscopy was applied on 14 strains during an outbreak with *E. cloacae* complex and provided fast typing results that were confirmed by WGS analysis. In conclusion, FTIR spectroscopy is a promising tool for strain typing of clinical *E. cloacae* complex strains. Discriminatory power can be improved by implementing an ANN for spectrum analysis. Due to its low costs and fast turnaround times, the method presents a valuable tool for real-time surveillance as well as outbreak analysis.

## Introduction

The *Enterobacter* (*E.*) *cloacae* complex comprises *E. cloacae* and several closely related species and subspecies that are difficult to differentiate by standard bacteriological identification methods such as Matrix-assisted laser desorption/ionization time-of-flight mass spectrometry (MALDI–TOF MS) or biochemical profiling (Davin-Regli et al., 2019). Nevertheless, members of the *E. cloacae* complex are highly relevant pathogens that are frequently involved in nosocomial infections such as urinary tract infections, blood stream infections, and sepsis. Correct species identification within the *E. cloacae* complex is desirable, because species might be differently able to cause infections in humans (Morand et al., 2009). Infections with these organisms especially affect predisposed patients like immune-compromised hosts and pre-term neonates (Hervas et al., 2001; Mezzatesta et al., 2012). Several outbreaks with *E. cloacae* have been reported from neonatal units in recent years (Dalben et al., 2008; Mezzatesta et al., 2012; Doijad et al., 2016), which suggests a high transmissibility of this pathogen in the hospital setting.

For outbreak analysis, fast and reliable strain typing methods of bacterial isolates are crucial to detect possible transmission routes of pathogens and to identify bacterial reservoirs (Li et al., 2009; Sabat et al., 2013). Due to their high discriminatory power, analysis methods that rely on whole genome sequencing (WGS) of bacterial isolates such as single-nucleotide polymorphism (SNP)-based phylogeny calculations and core genome multi-locus sequence typing (cgMLST) or whole genome MLST (wgMLST) are emerging as a gold standard for strain typing. However, these high-resolution techniques are still time consuming, have relatively high costs per sample, and require substantial computational resources, especially if many isolates must be compared. Thus, these techniques are often used retrospectively, but their application in real-time surveillance is still limited (Sabat et al., 2013; Salipante et al., 2015).

Fourier-transform infrared (FTIR) spectroscopy is a phenotypic method, which quantifies the absorption of infrared light by molecules such as lipopolysaccharides, lipids, carbohydrates, nucleic acids, and proteins, resulting in a specific FTIR spectrum that reflects the overall composition of the sample. The technique is widely used in analytical chemistry, but it was shown that it can also be applied to bacterial samples grown on solid or in liquid growth medium (Naumann et al., 1991). The resulting spectrum can be thought of as a specific “fingerprint” of a bacterial strain, which can be compared to other spectra by hierarchical or non-hierarchical clustering to investigate their similarity. Machine learning algorithms have also been used for this purpose (Grunert et al., 2013; Campos et al., 2018). The underlying assumption is that related strains are more similar in their composition (e.g., cell wall components), which leads to higher congruency of their FTIR spectra. Indeed, this principle has been used for genus and species identification of Gram-positive and Gram-negative bacteria (Wenning and Scherer, 2013; Quintelas et al., 2018). In some cases, FTIR spectroscopy was successfully applied for typing bacterial isolates on a sub-species level, which makes it an attractive tool for outbreak investigation. This has been demonstrated for human pathogens from environmental as well as from clinical sources, such as *Staphylococcus aureus* (Johler et al., 2016), *Escherichia coli* (Sousa et al., 2013), *Acinetobacter baumannii* complex (Sousa et al., 2014), and *Klebsiella* species (Dinkelacker et al., 2018) among others. Recently, the performance of FTIR spectroscopy was compared to conventional MLST for typing a collection of *E. cloacae* isolates (*n* = 23) recovered from outbreaks. The authors found a good separation of sequence types (STs) into distinct spectrum clusters (Martak et al., 2019).

The aim of the study presented here was to evaluate the discriminatory power of FTIR spectroscopy in a clinical setting on a larger set of *E. cloacae complex* isolates (*n* = 239), when all first isolates from patients as well as certain consecutive isolates (*n* = 53) were analyzed by a WGS approach. Furthermore, in addition to applying FTIR spectroscopy on this highly diverse set of collected strains, we employed the method on an unrelated set of isolates that were collected during a suspected outbreak of *E. cloacae* complex on the NICU of our hospital.

## Materials and Methods

### Strain Selection and Bacteriological Procedures

Anal and pharyngeal swabs are taken every week from all patients on the NICU of our hospital for screening of microbiological colonization according to German infection control guidelines. Screening samples as well as clinical samples used in this study were plated on Columbia sheep blood agar plates (Oxoid, Wesel, Germany), Endo agar (Oxoid, Wesel, Germany), or ESBL screening agar (bioMérieux, Marcy-l’Étoile, France). Interpretation was performed after incubation for 24 and 48 h at 37°C. Routine species identification was achieved by linear Matrix-assisted laser desorption/ionization time-of-flight mass spectrometry (MALDI–TOF MS) on a MALDI Biotyper system (based on a Microflex LT/SH instrument; Bruker Daltonik, Bremen, Germany). 239 isolates identified as *E. cloacae* complex from 24 patients were collected during a 4-month period (July 2017 until October 2017; Supplementary Table S1). Isolates were stored at −80°C until analysis. During a 5-week outbreak period (October 2018–November 2018) 14 additional isolates from 12 patients were collected (Supplementary Table S1) and analyzed immediately.

### FTIR Spectrum Acquisition and Analysis

Strains were grown at 37°C on Columbia sheep blood agar plates (Oxoid, Wesel, Germany) for 18 (±0.5) h. One inoculation loop full of bacteria was suspended in 50 μl of 70% (v/v) ethanol in 1.5 ml vials and thoroughly vortexed. After adding 50 μl of sterile H_2_O, 15 μl of the bacterial suspension was placed on a silicon sample plate (Bruker Daltonik, Bremen, Germany). Each isolate was analyzed in four technical replicates. The sample plate was dried at 37°C for approximately 20 min. The measurements were carried out using an IR Biotyper System (Bruker Daltonik, Bremen, Germany) running the IR Biotyper software (version 1.5) with the default analysis settings as recommended by the manufacturer. Measurements that did not meet the default quality criteria were excluded from further analysis. The second derivative of the 1,300–800 cm^–1^ wavenumber range of the spectra was calculated and used for further analysis, if not stated otherwise. After vector normalization and summarizing the replicate spectra, clustering was performed with the BioNumerics 7.6 software suite (Applied Maths, Sint-Martens-Latem, Belgium) using the unweighted pair group method with arithmetic mean (UPGMA) algorithm. Principal component analysis (PCA) was performed using the scikit-learn package^1^ (version 0.20.2) in Python (version 3.6.8).

### Genome Sequencing and Assembly

For WGS analysis, genomic DNA was extracted using the UltraClean Microbial DNA isolation kit (MOBIO Laboratories Inc., Carlsbad, CA, United States) or DNeasy Ultraclean Microbial Kit (Qiagen, Venlo, Netherlands). DNA libraries were prepared with the TruSeqNano DNA LT or HT Kit (Illumina, San Diego, CA, United States). Sequencing was performed on an Illumina MiSeq (Illumina, San Diego, CA, United States) or Illumina Nextseq (Illumina, San Diego, CA, United States) sequencer. Assembly of sequence reads was performed using the A5 pipeline (version 20140604) and SPAdes (version 3.7.0; Nurk et al., 2013; Coil et al., 2015).

### Genome Analysis

Core genomes for phylogenetic analysis were calculated using Spine (version 0.1.2; Ozer et al., 2014). Prophage regions were investigated using PHASTER^2^ (Arndt et al., 2016) and removed using a customized script of the A5 pipeline. SNP calling was performed by mapping high-quality sequencing reads previously generated by Trimmomatic (version 0.35; Bolger et al., 2014) to the core genome using BioNumerics 7.6 with default settings. Multi-locus STs (based on seven housekeeping genes) were extracted from the assembled sequences using the online MLST service for *E. cloacae* by the Center for Genomic Epidemiology^3^ (version 2.0) based on the MLST scheme for *E. cloacae* (Miyoshi-Akiyama et al., 2013). JSpecies (version 1.2; Richter et al., 2016) was used to calculate average nucleotide identity (ANI) for species identification based on the ANIm algorithm (Richter and Rossello-Mora, 2009). For this purpose, one genome of each SNP cluster was selected, and the ANI was calculated against two reference genomes of each species of the *E. cloacae* complex.

### Calculation of Clustering Concordance

The concordance of FTIR clustering in comparison to genotypic methods was determined by calculation of the adjusted rand index (ARI) (Carrico et al., 2006) using the online tool at www.comparingpartitions.info.

### Artificial Neural Network

The ANN was built using the Keras package^4^ (version 2.2.4) in Python (version 3.6.8) with a TensorFlow (version 1.12.0) backend. The ANN consisted of an input layer with 521 nodes (one node for each data point in the wavenumber range 1,300–800 cm^–1^), two hidden layers with 512 nodes each, and one output layer with two nodes for classification. A sigmoid activation function was used. To avoid overfitting, a dropout rate of 0.2 was chosen. The ANN was first trained and then applied to classify the difference between two FTIR spectra (“difference spectrum,” obtained by mathematical subtraction) to belong either to the same or different ST based on the output node value.

### Accession Numbers

The following genomes were used as references: *E. asburiae* ATCC35953 (NZ_CP011863.1), *E. asburiae* L1 (NZ_CP007546.1), *E. bugandensis* isolate EB-247 (NZ_LT992502.1), *E. bugandensis* IF3SW-P2 (NZ_POUO01000001.1), *E. ludwigii* P101 (NZ_CP006580.1), *E. ludwigii* EN-119 (NZ_CP017279.1), *E. roggenkampii* MGH132 (NZ_NGRL01000001.1), *E. roggenkampii* DSM 16690 (NZ_CP017184.1), *E. hormaechei* subsp. *steigerwaltii* strain DSM 16691 (NZ_CP017179.1), *E. hormaechei* subsp. *steigerwaltii* strain 34998 (NZ_CP012167.1), *E. hormaechei* subsp. *oharae* strain 34978 (NZ_CP012165.1), *E. hormaechei* subsp. *oharae* strain DSM 16687 (NZ_CP017180.1), *E. hormaechei* subsp. *hoffmannii* strain DSM 14563 (NZ_CP017186.1), and *E. hormaechei* subsp. *hoffmannii* strain AR 0365 (NZ_CP027142.1).

## Results

### WGS Analysis of *E. cloacae* Complex First Isolates

During a 4-month study period, all *E. cloacae* complex isolates (as identified by MALDI–TOF MS) from weekly microbiological screening (anal and pharyngeal swabs) or clinical specimens obtained from patients on the neonatal intensive care unit of our hospital were stored and used for further analysis. This resulted in a collection of 239 *E. cloacae* complex isolates (ID 1–239) from 24 patients (Supplementary Table S1).

**FIGURE 1:**
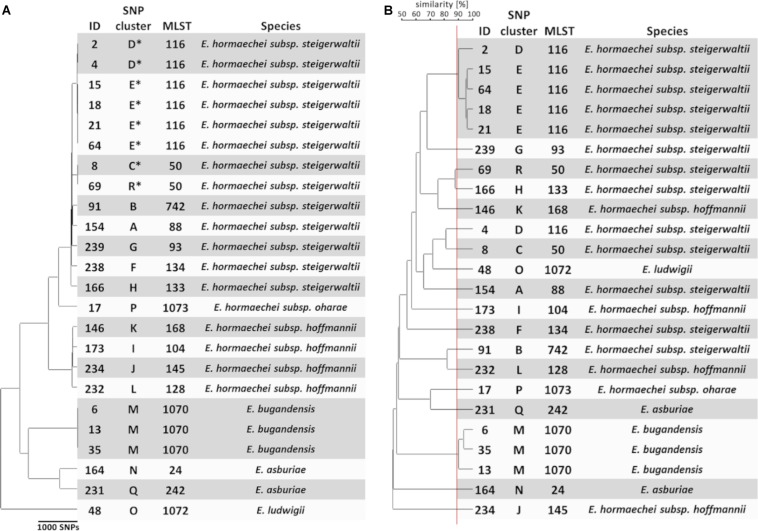
Genomic and spectral clustering of first *E. cloacae* complex isolates from 24 patients. **(A)** SNP-based phylogeny of *E. cloacae* complex isolates. SNP clusters that could be further delineated by SNP calling to sub-core genomes are indicated by an asterisk. The multi-locus sequence type (MLST) was extracted from the assembled genome sequences. Species identification was performed calculating the average nucleotide identity of one representative isolate from each SNP cluster compared to the reference genome. **(B)** Similarity cut-off value for FTIR spectrum clustering is shown as a vertical red line. SNP cluster, MLST, and species are derived from panel **(A)**.

To generate a reference for strain typing results obtained by FTIR spectroscopy, WGS was performed on the first isolate from each patient. Extraction of multi-locus STs from WGS data revealed the presence of 16 different ST in the collection (Figure 1A). Most ST were represented by a single isolate, whereas ST-50, ST-116, and ST-1070 comprised two, six, and three isolates, respectively. A single-nucleotide polymorphism (SNP)-based phylogeny of all 24 genomes was calculated using a core genome (2.8 Mbp size) (Figure 1A). For maximum resolution, a core genome was then built individually for each ST that was present in more than one isolate (ST-50, ST-116, and ST-1070). This led to the separation of ST-50 and ST-116 into two SNP clusters each (C/R and E/D), which differed by 776 and 2888 SNPs, respectively. However, genomes from ST-1070 (cluster M) could not be further delineated in this way, since they exhibited a maximum difference of two SNPs (Figure 1A). Altogether, the 24 *E. cloacae* complex isolates could be assigned to 18 SNP clusters (labeled “A” through “R”; Figure 1A), which suggests the presence of a substantial strain diversity found in our NICU patients during a 4-month period.

To assign a species to the 24 selected isolates, the ANI between the obtained whole genome sequences and two reference genomes for several species within the *E. cloacae* complex was determined. Four species (*E. hormaechei*, *E. asburiae*, *E. ludwigii*, and *E. bugandensis*) could be identified in our collection (Figure 1A and Supplementary Table S2). *E. hormaechei* isolates could be further delineated into three subspecies (*E. hormaechei* subsp. *steigerwaltii*, *E. hormaechei* subsp. *hoffmannii*, and *E. hormaechei* subsp. *oharae*) (Figure 1A and Supplementary Table S2). Interestingly, *E. cloacae* subsp. *cloacae* was not encountered in our samples.

### FTIR Spectroscopy of *E. cloacae* Complex Isolates

To test whether FTIR spectroscopy as a typing method is able to capture this genomic diversity, the technique was performed on all 24 isolates using four technical replicates in each of three independent measurements. Hierarchical clustering of the obtained summary spectra was then performed using the UPGMA method (Figure 1B). The ARI reflects the congruence of typing methods (Carrico et al., 2006), i.e., how well the groups or clusters obtained by two techniques “match” each other regarding their members. A value of 1.0 indicates complete concordance between clusters. The ARI was calculated for different similarity cut-off values from the obtained clustering in the dendrogram. The highest ARI values for comparison of FTIR spectrum-based and SNP-based clustering were 0.818, when a similarity cut-off value of 89% was chosen. Similarly, an ARI of 0.801 was calculated with a similarity cut-off value of 88% for the comparison for FTIR spectrum-based clustering and the ST of the isolates. These results indicate a high degree of similarity regarding the identification of related strains between the spectroscopic and the genomic methods (Figure 1B). Interestingly, both isolates belonging to ST-50 (SNP clusters C and R) were clearly separated by FTIR spectroscopy. Similarly, a separation of ST-116 isolates from SNP clusters D and E could be observed, when a high similarity cut-off value was chosen (Figure 1B). However, one isolate (ID 4; SNP cluster D; ST-116) was mistakenly separated from the other isolate (ID 2) of the same SNP cluster. Most of the isolates that belonged to different genomic entities showed a high Euclidean distance (i.e., low spectrum similarities) when compared to each other, but in some instances the similarity of the spectrum pairs was >80% (SNP clusters D and E, R and H, L and B, C and D), which suggests more similar composition of certain strains.

When the typing result obtained by FTIR spectroscopy was compared to the species and subspecies attribution, little congruency was observed (Figure 1B). For example, the four isolates (ID 146, 173, 232, and 234) identified as *E. hormaechei* subsp. *hoffmannii* were not grouped together by FTIR spectroscopy. Quantifying the concordance between FTIR clustering and species resulted in a maximum ARI of 0.630. The maximum ARI value was 0.567, when the subspecies was taken into account.

These results show that FTIR spectroscopy can discriminate many *E. cloacae* complex STs and is able to differentiate strains even within one ST.

### FTIR Spectroscopy of All 239 Isolates

**FIGURE 2:**
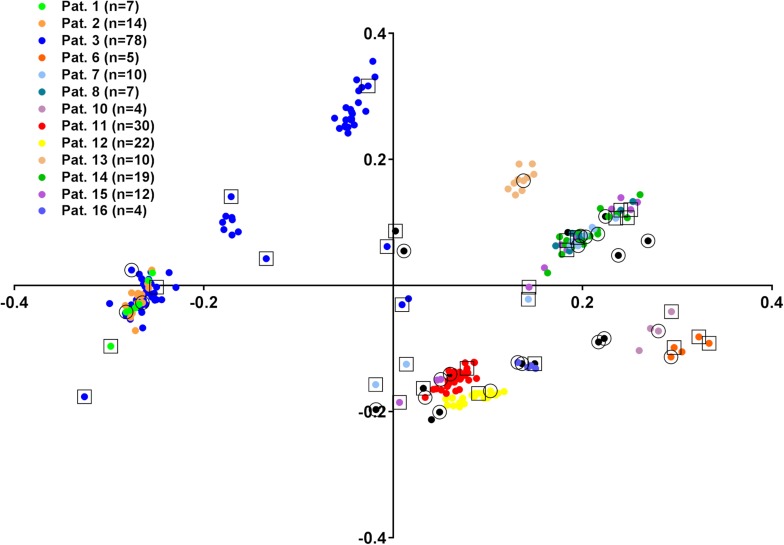
Principle component analysis (PCA) of 239 *E. cloacae* complex isolate FTIR spectra. Colors indicate isolates from the same patient, if more than two isolates were obtained. The number of isolates is given in the graph legend. Isolates from patients with less than three isolates (*n* = 2: patients 19, 20, 21, 22, 23, 24; *n* = 1: patients 4, 5, 9, 17, 18) are shown in black. First isolates are marked with an outer circle, additional isolates selected for WGS analysis are marked with an outer rectangle.

Next, we aimed to address the ability of FTIR spectroscopy to determine the relationship of consecutive isolates to the first isolate from one patient. For this purpose, the complete collection of 239 *E. cloacae* complex isolates was analyzed by FTIR spectroscopy in four technical replicates. The within-isolate similarity of the technical replicates was determined as 93.2% (±1.8% SD). A PCA was performed on all summary spectra to visualize their distribution (Figure 2). This indicated proximity of consecutive isolate spectra to the first isolate spectrum in many cases. However, some consecutive isolate spectra clustered separately, most prominent in patient 3, from which 78 isolates were gathered in total. This could indicate either colonization or co-colonization with a different *E. cloacae* complex strain or spectrum variability of the isolates over time. To resolve this question, consecutive isolates were chosen for WGS analysis (SNP-based phylogeny, MLST extraction, and species identification) and determination of genetic relationship with the first isolate from the same patient. For this purpose, a similarity matrix of all FTIR isolate spectra was calculated (Supplementary Table S3), and spectrum clusters were determined by assigning two spectra to the same cluster if their similarity was >91.4% (mean of technical replicate similarity minus one standard deviation). This led to the separation of all isolates into 42 groups (Supplementary Table S3), Eighteen of these groups contained at least one isolate that had been analyzed by WGS (see above). Another 29 isolates were then chosen that were also subjected to WGS with consecutive SNP analysis (Supplementary Figure S1) and species identification (Supplementary Table S2), so that the genomic typing information of at least one isolate per group was known. WGS analysis revealed that these isolates belonged to the same SNP cluster as the initial isolate from the same patient in all cases with one exception: patient 7 showed co-colonization with *E. hormaechei* subsp. *steigerwaltii* and *E. roggenkampii* (Supplementary Figure S1). Overall, these results indicate stable colonization of patients with one strain and considerable spectrum differences between isolates from the same SNP cluster. This was most prominent in isolates ID 103 and ID 144 from patient 3, which belonged to SNP cluster M, but had exhibited a spectrum similarity of only 44.7%.

**FIGURE 3:**
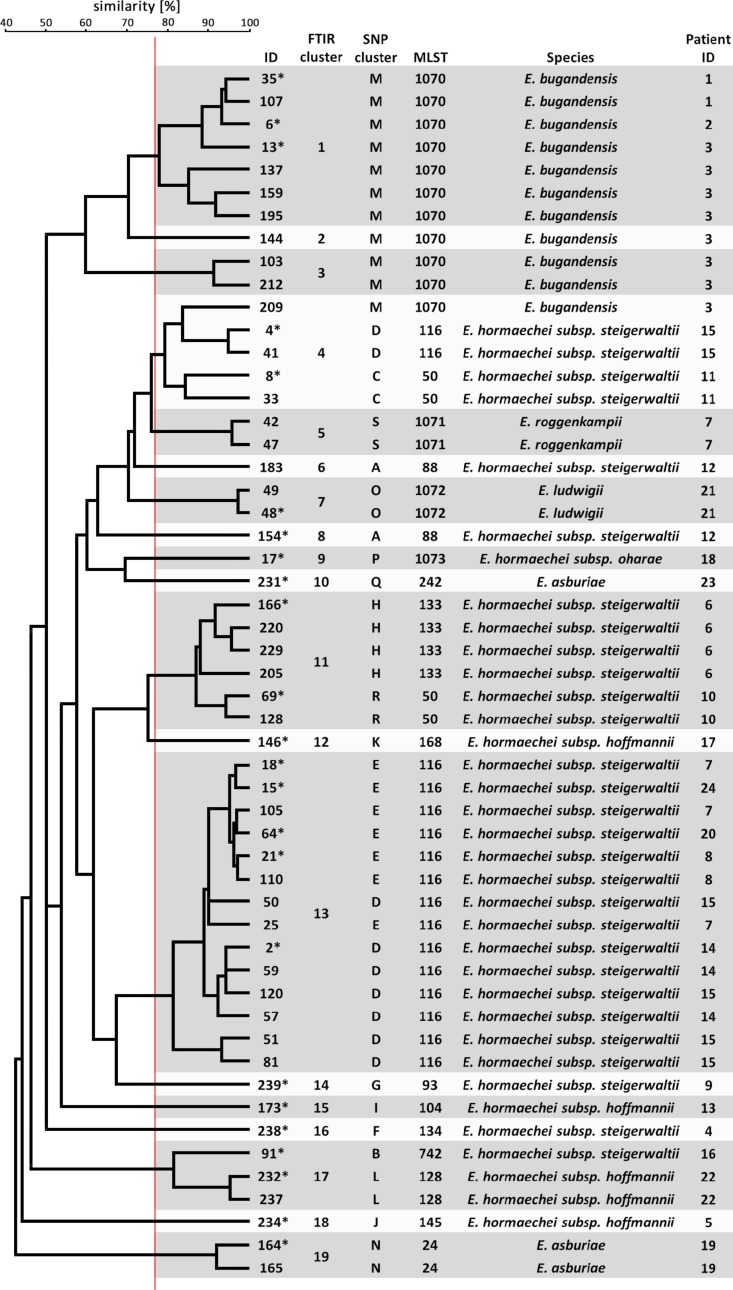
Clustering of FTIR spectra of 53 *E. cloacae* complex isolates. The dendrogram was calculated from the pairwise Euclidean distance of the isolate spectra by the UPGMA method. FTIR clusters are indicated as shaded boxes using a cut-off value of 77% similarity (red vertical line). SNP clusters were derived from the phylogenetic analysis (Supplementary Figure S2).

When an UPGMA clustering of the FTIR spectra of all 53 (24 first and 29 consecutive) sequenced isolates was performed the dendrogram in Figure 3 was obtained. The maximum ARI 0.716 was obtained for comparison of FTIR clustering and ST, when a similarity cut-off value of 77% was chosen. This is close to the cut-off value of 75% determined for *Klebsiella* spp. (Dinkelacker et al., 2018). The same similarity cut-off value of 77% yielded the maximum ARI of 0.527 for congruency of typing results by FTIR spectroscopy and phylogenetic SNP analysis. Ten of the resulting FTIR clusters contained isolates of only one ST. However, isolates belonging to ST-1070, ST-116, ST-50, and ST-88 were distributed over several FTIR clusters. Conversely, FTIR clusters 14, 6, and 2 contained isolates from different STs. When the similarity cut-off value was applied to all 1378 possible spectrum pairs, FTIR showed a sensitivity of 63.0% and a specificity of 97.0% to predict that two spectra belong to the same ST or not. The positive predictive value was 77.6%, whereas the negative predictive value was 94.2%.

These results demonstrate only moderate ability of FTIR spectroscopy to assign *E. cloacae* complex isolates to the same cluster, especially when many isolates from one patient are compared with each other. This may suggest spectrum variability within one genetic entity over time.

### Artificial Neural Network for Sequence Type Discrimination

Hierarchical clustering of the 53 isolate spectra by the UPGMA method resulted in only moderate discriminatory power for typing *E. cloacae* complex isolates in this study. It is conceivable that certain regions of the obtained spectra carry more information about a certain feature (e.g., ST), because these regions reflect different types of molecules, whereas other regions are more prone to intra- or inter-experimental variation without biological meaning. However, restricting the spectrum did not lead to a substantial improvement of clustering of *E. cloacae* complex strains (Supplementary Table S5).

Comparing two spectra (or parts of the acquired spectra) relies on the Euclidean distance between their data points, i.e., all data points are weighted equally. Therefore, we attempted to improve the discrimination and clustering of FTIR spectra in our dataset by using an artificial neural network (ANN), which has been described before for FTIR spectrum analysis of bacteria (Rebuffo et al., 2006; Grunert et al., 2013; Lasch et al., 2018).

**FIGURE 4:**
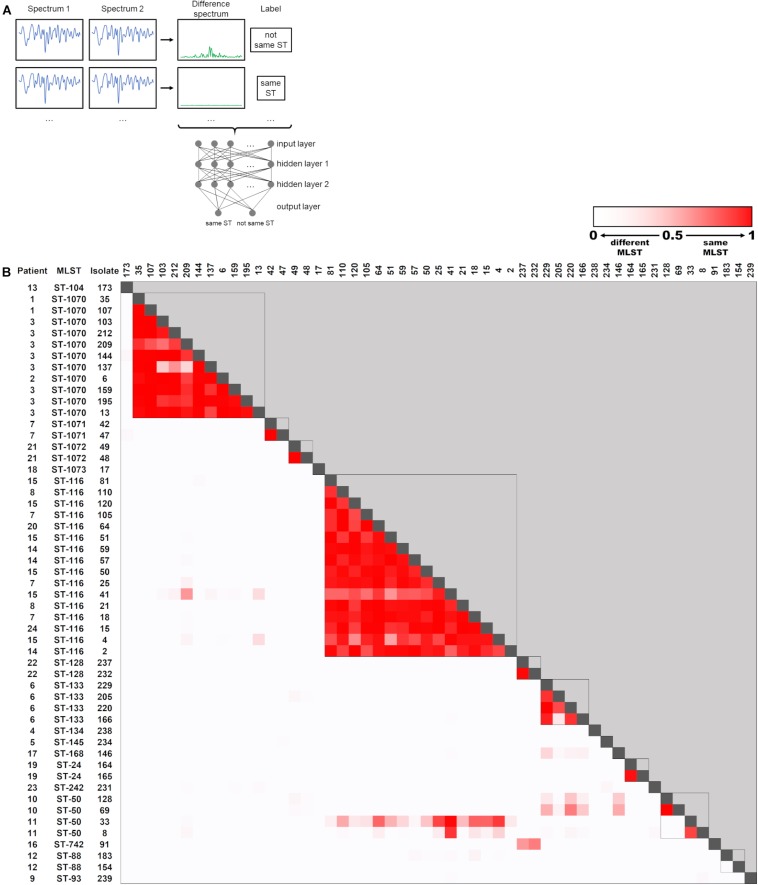
Artificial neural network (ANN) for determination of ST relationship of *E. cloacae* complex isolates. **(A)** Schematic representation of the ANN. Difference spectra are generated by subtraction of isolate summary spectra and fed into the ANN for training. Each datapoint of the spectrum (*n* = 521) corresponds to one input node. Output nodes are used for classification of the spectra, when the ANN is used for testing unknown spectrum pairs. **(B)** Heatmap of ANN output for classification of pairwise spectra as “same ST.” Spectrum pairs that were determined to belong to the same ST by WGS analysis are marked with a box.

During outbreak analysis, rapid typing methods should be able to quickly determine whether two isolates are of clonal origin, which would suggest a transmission event. Therefore, we trained the ANN to determine whether two spectra of the 53 sequenced isolates belonged to the same ST or not. For this purpose, a “difference spectrum” was calculated for every pair of isolate spectra by subtraction (Figure 4A). This resulted in a dataset containing 1378 “difference spectra” together with the binary information “same ST” or “not same ST” (Figure 4A). The dataset was split randomly into 10 parts, each of which contained 137 spectra (10% of the dataset) and was used as a test set after training the ANN with the remaining 90% of the dataset. One experiment consisted of 10 iterations of training/testing, so that every spectrum pair was once included in the test set without having been encountered by the ANN during training. Ten experiments were calculated to determine the average performance of the ANN.

The output of the ANN for classification as “same ST” is a value between 0 and 1, which can be interpreted as a “certainty” that two spectra belong to the same ST. These values are visualized for all FTIR spectrum pairs in Figure 4B. Overall, the ANN correctly identified 89.9% of all isolate pairs that belonged to the same ST (sensitivity) and 99.2% of all isolate pairs that did not belong to the same ST (specificity). Accordingly, the positive and negative predictive values were calculated as 95.1 and 98.3%, respectively.

These results indicate that employing an ANN can enhance the determination of relatedness of *E. cloacae* complex isolates compared to a clustering approach with a fixed similarity cut-off value.

### FTIR Spectroscopy for Outbreak Analysis

In October 2018 we observed a steep increase of the number of *E. cloacae* complex isolates detected on our neonatal intensive care unit, when the pathogen was recovered from specimens of 15 patients. While most patients were only colonized with the pathogen, one child developed a blood-stream infection.

**FIGURE 5:**
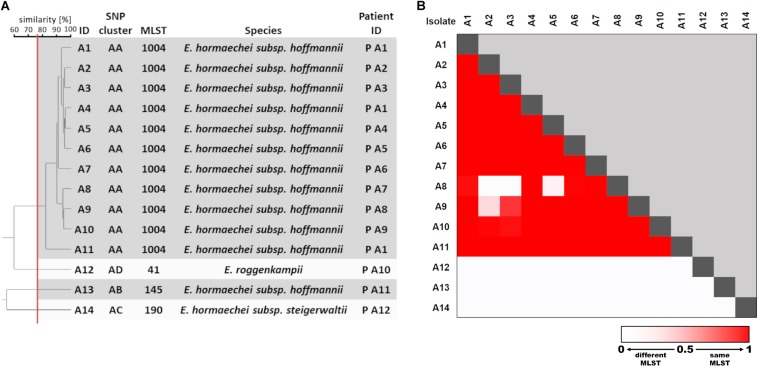
*E. cloacae* complex outbreak analysis with FTIR spectroscopy. **(A)** UPGMA clustering of FTIR spectra of 14 Enterobacter cloacae complex isolates. SNP cluster attribution was based on phylogenetic analysis (Supplementary Figure S2). MLST and species were extracted from WGS data. FTIR clusters are indicated as shaded boxes using a cut-off value of 77% similarity (red vertical line). **(B)** Heatmap of ANN output for classification of pairwise spectra as “same ST.”

Fourier-transform infrared spectroscopy was performed for rapid outbreak analysis on 14 available isolates obtained from 12 patients (“A1” through “A12) during a 5-week period. All first bacterial isolates from screening swabs of each patient as well as clinical isolates from patient A1 (blood culture, central venous catheter) were included (ID “A1” through “A14”; Supplementary Table S1). Each isolate was measured in quadruplicates in three independent experiments. UPGMA clustering of FTIR isolate spectra using the established 77% cut-off similarity value revealed one large cluster comprising 11 isolates and 3 single isolates (Figure 5A). One isolate (“A11”) from the large cluster exhibited a considerably greater spectroscopic distance from the other cluster isolates (Figure 5A). After subjection of all 14 isolates to WGS, SNP analysis based on a core genome (size 4.0 Mbp) confirmed the results obtained by FTIR spectroscopy and revealed four SNP clusters, which represented four different STs (Supplementary Figure S2). Using the trained ANN led to the same overall result (Figure 5B). Interestingly, the ANN falsely detected differences between spectra A8 and A2, A3, and A5 as well as between spectra A9 and A2, which were not apparent in the UPGMA clustering (Figure 5A). This demonstrates that caution is warranted when machine learning algorithms are used for classification. The species identification using the ANI method revealed that the large cluster belonged to *E. hormaechei* subsp. *hoffmannii*, whereas the three single isolates belonged to a different ST of *E. hormaechei* subsp. *hoffmannii*, *E. hormaechei* subsp. *steigerwaltii*, and *E. roggenkampii* (Supplementary Table S4). Interestingly there was no overlap of STs from strains obtained from the same NICU 1 year previously.

These results show that FTIR spectroscopy can be a valuable tool for rapid outbreak analysis.

## Discussion

In this study the performance of FTIR spectroscopy as a rapid strain typing method for clinical *E. cloacae* complex isolates was evaluated. While other studies that employed this technique for rapid identification or typing of several bacterial species mostly relied on predefined collections of strains, here, clinical isolates gathered during routine microbiological surveillance and during an outbreak with *E. cloacae* complex on a neonatal intensive care unit were employed to mimic a situation when the quick determination of the clonality of the isolates is desirable.

We found that FTIR spectroscopy is able to capture the diversity of *E. cloacae* strains in an endemic situation, which is overlooked, when only MALDI–TOF MS is used for species identification. This is reflected by an ARI of 0.801 and 0.818 when FTIR spectroscopy was used on 24 first isolates and was compared to MLST- and SNP-based phylogeny, respectively. This is in line with another study that compared FTIR spectroscopy and MLST (Martak et al., 2019). The results obtained for *E. cloacae* complex are comparable to a similar typing approach in *Klebsiella* spp. (Dinkelacker et al., 2018). Another study also demonstrated the capability of FTIR spectroscopy to distinguish different *A. baumannii* ST (Sousa et al., 2014). A recent report describes the successful delineation of *Salmonella* serogroups and some serotypes by this technique in a large collection containing 325 isolates (Campos et al., 2018). The recovery of genetically related strains in our study from screening specimens of different patients suggests that transmission of bacteria occurs in some instances in the intensive care setting.

Our results also show that the spectroscopic distance is largely independent of the genetic distance of two *E. cloacae complex* strains, since FTIR-based clustering of the isolates showed little congruence with species or subspecies attribution. Several studies point toward the structural variation of the O-antigen as the underlying reason for the ability of FTIR spectroscopy to discriminate strains on an infra-species level. This is supported by the fact that the carbohydrate region of the spectrum is important for differentiating *Yersinia enterocolitica* biotypes and serotypes (Kuhm et al., 2009). This was also the case in *Campylobacter* spp., when then polysaccharide region (1,200–900 cm^–1^) spectral range allowed discrimination of 17 strains (Mouwen et al., 2005). Studies performed on *E. coli* indicated that O-antigens O4 and O123 can be well distinguished by FTIR spectroscopy, when the same spectral range was used (Beutin et al., 2007). On the other hand, six *E. coli* serotypes could be separated by this technique, when outer membrane preparations and a spectral range of 1,800–1,500 cm^–1^ were employed (Kim et al., 2006). In organisms that lack the lipopolysaccharide of the Gram-negative cell wall such as *S. aureus* or *Streptococcus pneumoniae*, other cellular structures such as the capsule are the basis for successful strain delineation by FTIR spectroscopy (Sahu et al., 2006). For *E. cloacae* complex strains, we found highest concordance with genetic clustering when the polysaccharide region (1,200–900 cm^–1^) and the fingerprint region (900–700 cm^–1^) were used for spectrum analysis.

Apart from 24 first isolates, our collection also comprised 215 subsequent *E. cloacae* complex strains from the same patients. 105 of these isolates were highly similar to the first isolates, which shows stable colonization of the patients with one strain most of the time. Isolates that showed less similarity to first isolates were subjected to WGS and genetic identity with the first isolates was established in all cases but one. When all sequenced isolates were included in a hierarchical clustering of FTIR spectra, the ARI was lower compared to using only the first isolates. However, one has to keep in mind that this selection represented the most diverse isolates. But this also shows that considerable variability of FTIR spectra of following isolates can occur. Since the FTIR spectrum reflects the chemical composition of the analyzed bacterial cells, constant growth conditions (incubation time and temperature, growth medium) are mandatory, when comparing isolate spectra with each other. Since these factors were kept constant in our study, other factors that influence cell composition (e.g., regulation of cell wall synthesis) might be responsible for the observed variability.

The choice of the similarity cut-off value has an obvious influence on the clustering of FTIR spectra and thereby the performance of the method in comparison to a reference technique (Martak et al., 2019). We therefore applied an ANN to spectrum analysis to improve the discriminatory power of FTIR spectroscopy in our isolate collection. Supervised machine learning techniques such as ANNs can be employed for data classification and have been used on microbiological FTIR spectrum datasets before. This approach was used to reliably discriminate *S. aureus* capsular serotypes 5, 8, and non-typeable isolates (Grunert et al., 2013) or to identify various *Listeria* species (Rebuffo et al., 2006). In a recent study, a hierarchical ANN architecture was used to correctly identify a diverse set of Gram-positive and Gram-negative human pathogens as well as a collection of closely related *Burkholderia* spp. strains (Lasch et al., 2018). The performance of ANNs to achieve the task of attributing a given FTIR spectrum to a certain group (e.g., genus, species, serotype) is highly dependent on the size and comprehensiveness of the training data set, which is often limited when dealing with bacterial isolate collections. However, in certain situations such as outbreak investigations it might be sufficient to know whether two strains recovered from clinical samples belong to the same entity (e.g., serotype, ST) or not. Therefore, we developed an ANN in our study that was trained to recognize if two isolates belong to the same ST assuming that this information can be extracted from the difference between the two FTIR spectra. This enhances the approach of simply calculating the Euclidean distance between two spectra, because the ANN is able to weigh certain parts of the spectrum differently. Moreover, the amount of training data is vastly increased as *N* spectra will yield *N* × (*N*−1)/2 spectrum pairs. We could show that this method can indeed improve the recognition of *E. cloacae* complex isolates from the same or distinct STs. However, one has to keep in mind that training and employing ANNs is a challenge for the clinical laboratory. It would be desirable that manufacturers implement this feature in the analysis software of the instrument to increase its applicability. One prerequisite for this would be the construction of large spectrum databases that cover a broad and – most importantly – well characterized collection of isolates of a species.

Few studies exist that used FTIR spectroscopy for strain typing during outbreak investigation. In one report this technique was used for isolate characterization of *Klebsiella oxytoca* from contaminated liquid hand soap with similar resolution as WGS-based methods (Dieckmann et al., 2016). Martak et al. (2019) found that when FTIR spectroscopy was used on *E. cloacae* isolates gathered during several outbreaks and analyzed retrospectively, the results were similar to MLST typing. The increased occurrence of one bacterial species from patient specimens in an epidemiological setting (e.g., on one ward) warrants outbreak investigations and possibly the implementation of transmission precautions. FTIR spectroscopy can be of help to determine if the isolates are likely to be clonally related, or if several unrelated strains are causing a pseudo-outbreak.

Overall, FTIR spectroscopy proves to be a promising tool for fast and cost-effective strain typing in the clinical microbiology laboratory during routine pathogen surveillance as well as in outbreak situations.

## Data Availability Statement

The assembled genomes of the isolates used in this study are available in the NCBI GenBank repository under the project accession number PRJNA554224.

## Ethics Statement

Bacterial isolates used in this study were collected from routine microbiological specimens obtained according to the German infection control guidelines and were anonymized. Outbreak investigation was mandated under the German Infection Protection Act (Infektionsschutzgesetz). Patients were not physically involved in this study. According to the professional code of conduct of the Baden-Württemberg Medical Association (Landesärztekammer), approval from an ethics committee is therefore not required.

## Author Contributions

SV and JL conceived the study and wrote the manuscript with critical input from all authors. SV, KL, AD, and BB performed the experiments. SV, AD, IA, SP, and JL analyzed the data.

## Conflict of Interest

The authors declare that the research was conducted in the absence of any commercial or financial relationships that could be construed as a potential conflict of interest.

## Footnotes

1 https://scikit-learn.org/

2 https://phaster.ca

3 https://cge.cbs.dtu.dk/services/MLST/

4 https://keras.io
