# Supplementary material for: Fourier-Transform Infrared (FTIR) Spectroscopy for Typing of Clinical Enterobacter cloacae Complex Isolates
Source: Front Microbiol. 2019 Nov 6;10:2582. doi: 10.3389/fmicb.2019.02582 (PMC6851243; doi:10.3389/fmicb.2019.02582)

Table S1: Characteristics of clinical *E. cloacae* complex isolates collected for routine microbiology diagnostic purpose or during an outbreak. The first isolate from each patient as well as isolates analyzed by whole-genome sequencing (WGS) are marked with an “X” in the respective column.

| **Isolate ID** | **Collection date (Month-Year)** | **Reason for collection** | **Patient ID** | **Source** | **First isolate** | **WGS** |
| --- | --- | --- | --- | --- | --- | --- |
| 1 | 07-2017 | Routine | 14 | pharyngeal swab |  |  |
| 2 | 07-2017 | Routine | 14 | anal swab | X | X |
| 3 | 07-2017 | Routine | 14 | anal swab |  |  |
| 4 | 07-2017 | Routine | 15 | pharyngeal swab | X | X |
| 5 | 07-2017 | Routine | 15 | anal swab |  |  |
| 6 | 07-2017 | Routine | 2 | pharyngeal swab | X | X |
| 7 | 07-2017 | Routine | 2 | anal swab |  |  |
| 8 | 07-2017 | Routine | 11 | anal swab | X | X |
| 9 | 07-2017 | Routine | 14 | pharyngeal swab |  |  |
| 10 | 07-2017 | Routine | 14 | pharyngeal swab |  |  |
| 11 | 07-2017 | Routine | 2 | anal swab |  |  |
| 12 | 07-2017 | Routine | 11 | anal swab |  |  |
| 13 | 07-2017 | Routine | 3 | pharyngeal swab | X | X |
| 14 | 07-2017 | Routine | 3 | pharyngeal swab |  |  |
| 15 | 07-2017 | Routine | 24 | pharyngeal swab | X | X |
| 16 | 07-2017 | Routine | 24 | anal swab |  |  |
| 17 | 07-2017 | Routine | 18 | anal swab | X | X |
| 18 | 07-2017 | Routine | 7 | pharyngeal swab | X | X |
| 19 | 07-2017 | Routine | 7 | anal swab |  |  |
| 20 | 07-2017 | Routine | 8 | anal swab |  |  |
| 21 | 07-2017 | Routine | 8 | pharyngeal swab | X | X |
| 22 | 07-2017 | Routine | 8 | anal swab |  |  |
| 23 | 07-2017 | Routine | 3 | Intra-operative swab |  |  |
| 24 | 07-2017 | Routine | 3 | skin swab |  |  |
| 25 | 07-2017 | Routine | 7 | anal swab |  | X |
| 26 | 07-2017 | Routine | 3 | pharyngeal swab |  |  |
| 27 | 07-2017 | Routine | 20 | anal swab |  |  |
| 28 | 07-2017 | Routine | 8 | pharyngeal swab |  |  |
| 29 | 07-2017 | Routine | 2 | pharyngeal swab |  |  |
| 30 | 07-2017 | Routine | 14 | pharyngeal swab |  |  |
| 31 | 07-2017 | Routine | 14 | anal swab |  |  |
| 32 | 07-2017 | Routine | 14 | anal swab |  |  |
| 33 | 07-2017 | Routine | 11 | anal swab |  | X |
| 34 | 07-2017 | Routine | 14 | pharyngeal swab |  |  |
| 35 | 07-2017 | Routine | 1 | anal swab | X | X |
| 36 | 07-2017 | Routine | 2 | anal swab |  |  |
| 37 | 07-2017 | Routine | 7 | pharyngeal swab |  |  |
| 38 | 07-2017 | Routine | 8 | anal swab |  |  |
| 39 | 07-2017 | Routine | 3 | anal swab |  |  |
| 40 | 07-2017 | Routine | 3 | anal swab |  |  |
| 41 | 07-2017 | Routine | 15 | anal swab |  | X |
| 42 | 07-2017 | Routine | 7 | anal swab |  | X |
| 43 | 07-2017 | Routine | 2 | pharyngeal swab |  |  |
| 44 | 07-2017 | Routine | 11 | anal swab |  |  |
| 45 | 07-2017 | Routine | 3 | pharyngeal swab |  |  |
| 46 | 07-2017 | Routine | 2 | pharyngeal swab |  |  |
| 47 | 07-2017 | Routine | 7 | anal swab |  | X |
| 48 | 07-2017 | Routine | 21 | pharyngeal swab | X | X |
| 49 | 07-2017 | Routine | 21 | anal swab |  | X |
| 50 | 07-2017 | Routine | 15 | pharyngeal swab |  | X |
| 51 | 07-2017 | Routine | 15 | anal swab |  | X |
| 52 | 07-2017 | Routine | 11 | anal swab |  |  |
| 53 | 07-2017 | Routine | 3 | anal swab |  |  |
| 54 | 07-2017 | Routine | 3 | anal swab |  |  |
| 55 | 07-2017 | Routine | 3 | pharyngeal swab |  |  |
| 56 | 07-2017 | Routine | 3 | wound secretion |  |  |
| 57 | 07-2017 | Routine | 14 | anal swab |  | X |
| 58 | 07-2017 | Routine | 14 | pharyngeal swab |  |  |
| 59 | 07-2017 | Routine | 14 | anal swab |  | X |
| 60 | 07-2017 | Routine | 1 | anal swab |  |  |
| 61 | 07-2017 | Routine | 1 | pharyngeal swab |  |  |
| 62 | 07-2017 | Routine | 2 | pharyngeal swab |  |  |
| 63 | 07-2017 | Routine | 2 | anal swab |  |  |
| 64 | 07-2017 | Routine | 20 | anal swab | X | X |
| 65 | 07-2017 | Routine | 11 | anal swab |  |  |
| 66 | 07-2017 | Routine | 11 | anal swab |  |  |
| 67 | 07-2017 | Routine | 3 | pharyngeal swab |  |  |
| 68 | 07-2017 | Routine | 3 | pharyngeal swab |  |  |
| 69 | 07-2017 | Routine | 10 | anal swab | X | X |
| 70 | 07-2017 | Routine | 7 | anal swab |  |  |
| 71 | 07-2017 | Routine | 3 | wound swab |  |  |
| 72 | 07-2017 | Routine | 3 | wound swab |  |  |
| 73 | 07-2017 | Routine | 3 | intraperitoneal swab |  |  |
| 74 | 07-2017 | Routine | 3 | intraperitoneal swab |  |  |
| 75 | 07-2017 | Routine | 11 | pharyngeal swab |  |  |
| 76 | 07-2017 | Routine | 11 | anal swab |  |  |
| 77 | 07-2017 | Routine | 11 | anal swab |  |  |
| 78 | 07-2017 | Routine | 11 | anal swab |  |  |
| 79 | 07-2017 | Routine | 3 | pharyngeal swab |  |  |
| 80 | 07-2017 | Routine | 15 | pharyngeal swab |  |  |
| 81 | 07-2017 | Routine | 15 | anal swab |  | X |
| 82 | 07-2017 | Routine | 16 | nasal swab |  |  |
| 83 | 07-2017 | Routine | 16 | pharyngeal swab |  |  |
| 84 | 07-2017 | Routine | 3 | pharyngeal swab |  |  |
| 85 | 07-2017 | Routine | 3 | anal swab |  |  |
| 86 | 07-2017 | Routine | 3 | anal swab |  |  |
| 87 | 07-2017 | Routine | 3 | anal swab |  |  |
| 88 | 07-2017 | Routine | 3 | anal swab |  |  |
| 89 | 07-2017 | Routine | 15 | anal swab |  |  |
| 90 | 07-2017 | Routine | 16 | nasal swab |  |  |
| 91 | 07-2017 | Routine | 16 | anal swab | X | X |
| 92 | 08-2017 | Routine | 14 | anal swab |  |  |
| 93 | 08-2017 | Routine | 14 | pharyngeal swab |  |  |
| 94 | 08-2017 | Routine | 14 | pharyngeal swab |  |  |
| 95 | 07-2017 | Routine | 7 | anal swab |  |  |
| 96 | 07-2017 | Routine | 7 | anal swab |  |  |
| 97 | 08-2017 | Routine | 10 | anal swab |  |  |
| 98 | 08-2017 | Routine | 1 | pharyngeal swab |  |  |
| 99 | 08-2017 | Routine | 2 | pharyngeal swab |  |  |
| 100 | 08-2017 | Routine | 2 | anal swab |  |  |
| 101 | 07-2017 | Routine | 3 | wound swab |  |  |
| 102 | 07-2017 | Routine | 3 | wound swab |  |  |
| 103 | 07-2017 | Routine | 3 | pharyngeal swab |  | X |
| 104 | 08-2017 | Routine | 14 | anal swab |  |  |
| 105 | 07-2017 | Routine | 7 | pharyngeal swab |  | X |
| 106 | 08-2017 | Routine | 1 | anal swab |  |  |
| 107 | 08-2017 | Routine | 1 | pharyngeal swab |  | X |
| 108 | 08-2017 | Routine | 2 | anal swab |  |  |
| 109 | 07-2017 | Routine | 8 | anal swab |  |  |
| 110 | 07-2017 | Routine | 8 | pharyngeal swab |  | X |
| 111 | 07-2017 | Routine | 3 | anal swab |  |  |
| 112 | 07-2017 | Routine | 3 | anal swab |  |  |
| 113 | 08-2017 | Routine | 3 | anal swab |  |  |
| 114 | 08-2017 | Routine | 3 | pharyngeal swab |  |  |
| 115 | 08-2017 | Routine | 3 | pharyngeal swab |  |  |
| 116 | 08-2017 | Routine | 3 | pharyngeal swab |  |  |
| 117 | 08-2017 | Routine | 11 | pharyngeal swab |  |  |
| 118 | 08-2017 | Routine | 14 | anal swab |  |  |
| 119 | 08-2017 | Routine | 14 | pharyngeal swab |  |  |
| 120 | 08-2017 | Routine | 15 | anal swab |  | X |
| 121 | 08-2017 | Routine | 15 | pharyngeal swab |  |  |
| 122 | 08-2017 | Routine | 15 | pharyngeal swab |  |  |
| 123 | 08-2017 | Routine | 15 | anal swab |  |  |
| 124 | 08-2017 | Routine | 3 | anal swab |  |  |
| 125 | 08-2017 | Routine | 3 | anal swab |  |  |
| 126 | 08-2017 | Routine | 3 | pharyngeal swab |  |  |
| 127 | 08-2017 | Routine | 11 | anal swab |  |  |
| 128 | 08-2017 | Routine | 10 | anal swab |  | X |
| 129 | 08-2017 | Routine | 2 | anal swab |  |  |
| 130 | 08-2017 | Routine | 14 | pharyngeal swab |  |  |
| 131 | 08-2017 | Routine | 1 | anal swab |  |  |
| 132 | 08-2017 | Routine | 3 | anal swab |  |  |
| 133 | 08-2017 | Routine | 3 | anal swab |  |  |
| 134 | 08-2017 | Routine | 11 | anal swab |  |  |
| 135 | 08-2017 | Routine | 11 | anal swab |  |  |
| 136 | 08-2017 | Routine | 2 | pharyngeal swab |  |  |
| 137 | 08-2017 | Routine | 3 | anal swab |  | X |
| 138 | 08-2017 | Routine | 11 | pharyngeal swab |  |  |
| 139 | 08-2017 | Routine | 3 | tracheal aspirate |  |  |
| 140 | 08-2017 | Routine | 11 | pharyngeal swab |  |  |
| 141 | 08-2017 | Routine | 3 | pharyngeal swab |  |  |
| 142 | 08-2017 | Routine | 11 | anal swab |  |  |
| 143 | 08-2017 | Routine | 10 | anal swab |  |  |
| 144 | 08-2017 | Routine | 3 | wound swab |  | X |
| 145 | 08-2017 | Routine | 11 | pharyngeal swab |  |  |
| 146 | 08-2017 | Routine | 17 | nasal swab | X | X |
| 147 | 08-2017 | Routine | 3 | conjunctival swab |  |  |
| 148 | 08-2017 | Routine | 11 | pharyngeal swab |  |  |
| 149 | 08-2017 | Routine | 3 | pharyngeal swab |  |  |
| 150 | 08-2017 | Routine | 11 | pharyngeal swab |  |  |
| 151 | 08-2017 | Routine | 11 | anal swab |  |  |
| 152 | 08-2017 | Routine | 3 | pharyngeal swab |  |  |
| 153 | 08-2017 | Routine | 3 | pharyngeal swab |  |  |
| 154 | 08-2017 | Routine | 12 | anal swab | X | X |
| 155 | 08-2017 | Routine | 11 | tracheal aspirate |  |  |
| 156 | 08-2017 | Routine | 3 | pharyngeal swab |  |  |
| 157 | 08-2017 | Routine | 3 | pharyngeal swab |  |  |
| 158 | 08-2017 | Routine | 11 | pharyngeal swab |  |  |
| 159 | 08-2017 | Routine | 3 | anal swab |  | X |
| 160 | 08-2017 | Routine | 3 | anal swab |  |  |
| 161 | 08-2017 | Routine | 3 | anal swab |  |  |
| 162 | 08-2017 | Routine | 3 | anal swab |  |  |
| 163 | 08-2017 | Routine | 3 | pharyngeal swab |  |  |
| 164 | 08-2017 | Routine | 19 | anal swab | X | X |
| 165 | 08-2017 | Routine | 19 | anal swab |  | X |
| 166 | 08-2017 | Routine | 6 | anal swab | X | X |
| 167 | 08-2017 | Routine | 12 | pharyngeal swab |  |  |
| 168 | 08-2017 | Routine | 12 | anal swab |  |  |
| 169 | 08-2017 | Routine | 11 | tracheal aspirate |  |  |
| 170 | 08-2017 | Routine | 11 | anal swab |  |  |
| 171 | 09-2017 | Routine | 11 | pharyngeal swab |  |  |
| 172 | 09-2017 | Routine | 12 | pharyngeal swab |  |  |
| 173 | 09-2017 | Routine | 13 | anal swab | X | X |
| 174 | 09-2017 | Routine | 13 | pharyngeal swab |  |  |
| 175 | 09-2017 | Routine | 3 | pharyngeal swab |  |  |
| 176 | 09-2017 | Routine | 3 | pharyngeal swab |  |  |
| 177 | 09-2017 | Routine | 3 | pharyngeal swab |  |  |
| 178 | 09-2017 | Routine | 12 | anal swab |  |  |
| 179 | 09-2017 | Routine | 6 | anal swab |  |  |
| 180 | 09-2017 | Routine | 3 | pharyngeal swab |  |  |
| 181 | 09-2017 | Routine | 12 | anal swab |  |  |
| 182 | 09-2017 | Routine | 12 | anal swab |  |  |
| 183 | 09-2017 | Routine | 12 | anal swab |  | X |
| 184 | 09-2017 | Routine | 12 | pharyngeal swab |  |  |
| 185 | 09-2017 | Routine | 3 | anal swab |  |  |
| 186 | 09-2017 | Routine | 11 | pharyngeal swab |  |  |
| 187 | 09-2017 | Routine | 3 | nasal swab |  |  |
| 188 | 09-2017 | Routine | 3 | tracheal aspirate |  |  |
| 189 | 09-2017 | Routine | 12 | anal swab |  |  |
| 190 | 09-2017 | Routine | 12 | pharyngeal swab |  |  |
| 191 | 09-2017 | Routine | 3 | pharyngeal swab |  |  |
| 192 | 09-2017 | Routine | 3 | pharyngeal swab |  |  |
| 193 | 09-2017 | Routine | 3 | pharyngeal swab |  |  |
| 194 | 09-2017 | Routine | 3 | anal swab |  |  |
| 195 | 09-2017 | Routine | 3 | anal swab |  | X |
| 196 | 09-2017 | Routine | 12 | pharyngeal swab |  |  |
| 197 | 09-2017 | Routine | 13 | pharyngeal swab |  |  |
| 198 | 09-2017 | Routine | 11 | pharyngeal swab |  |  |
| 199 | 09-2017 | Routine | 11 | pharyngeal swab |  |  |
| 200 | 09-2017 | Routine | 3 | anal swab |  |  |
| 201 | 09-2017 | Routine | 12 | pharyngeal swab |  |  |
| 202 | 09-2017 | Routine | 3 | anal swab |  |  |
| 203 | 09-2017 | Routine | 12 | anal swab |  |  |
| 204 | 09-2017 | Routine | 13 | anal swab |  |  |
| 205 | 09-2017 | Routine | 6 | anal swab |  | X |
| 206 | 09-2017 | Routine | 13 | pharyngeal swab |  |  |
| 207 | 09-2017 | Routine | 3 | pharyngeal swab |  |  |
| 208 | 09-2017 | Routine | 3 | anal swab |  |  |
| 209 | 09-2017 | Routine | 3 | pharyngeal swab |  | X |
| 210 | 09-2017 | Routine | 3 | anal swab |  |  |
| 211 | 09-2017 | Routine | 3 | anal swab |  |  |
| 212 | 09-2017 | Routine | 3 | pharyngeal swab |  | X |
| 213 | 09-2017 | Routine | 12 | pharyngeal swab |  |  |
| 214 | 09-2017 | Routine | 12 | anal swab |  |  |
| 215 | 09-2017 | Routine | 13 | pharyngeal swab |  |  |
| 216 | 09-2017 | Routine | 3 | secretion |  |  |
| 217 | 09-2017 | Routine | 12 | pharyngeal swab |  |  |
| 218 | 09-2017 | Routine | 13 | pharyngeal swab |  |  |
| 219 | 09-2017 | Routine | 3 | tracheal aspirate |  |  |
| 220 | 09-2017 | Routine | 6 | anal swab |  | X |
| 221 | 09-2017 | Routine | 12 | anal swab |  |  |
| 222 | 09-2017 | Routine | 13 | anal swab |  |  |
| 223 | 09-2017 | Routine | 3 | pharyngeal swab |  |  |
| 224 | 09-2017 | Routine | 12 | pharyngeal swab |  |  |
| 225 | 09-2017 | Routine | 3 | pharyngeal swab |  |  |
| 226 | 09-2017 | Routine | 3 | pharyngeal swab |  |  |
| 227 | 09-2017 | Routine | 12 | anal swab |  |  |
| 228 | 09-2017 | Routine | 12 | anal swab |  |  |
| 229 | 09-2017 | Routine | 6 | anal swab |  | X |
| 230 | 09-2017 | Routine | 13 | anal swab |  |  |
| 231 | 10-2017 | Routine | 23 | anal swab | X | X |
| 232 | 10-2017 | Routine | 22 | anal swab | X | X |
| 233 | 10-2017 | Routine | 23 | anal swab |  |  |
| 234 | 10-2017 | Routine | 5 | pharyngeal swab | X | X |
| 235 | 10-2017 | Routine | 12 | pharyngeal swab |  |  |
| 236 | 10-2017 | Routine | 13 | anal swab |  |  |
| 237 | 10-2017 | Routine | 22 | anal swab |  | X |
| 238 | 10-2017 | Routine | 4 | anal swab | X | X |
| 239 | 10-2017 | Routine | 9 | pharyngeal swab | X | X |
| A1 | 10-2018 | Outbreak | A1 | pharyngeal swab | X | X |
| A2 | 10-2018 | Outbreak | A2 | anal swab | X | X |
| A3 | 10-2018 | Outbreak | A3 | anal swab | X | X |
| A4 | 10-2018 | Outbreak | A1 | blood culture |  | X |
| A5 | 10-2018 | Outbreak | A4 | anal swab | X | X |
| A6 | 10-2018 | Outbreak | A5 | anal swab | X | X |
| A7 | 10-2018 | Outbreak | A6 | anal swab | X | X |
| A8 | 10-2018 | Outbreak | A7 | pharyngeal swab | X | X |
| A9 | 10-2018 | Outbreak | A8 | anal swab | X | X |
| A10 | 11-2018 | Outbreak | A9 | anal swab | X | X |
| A11 | 10-2018 | Outbreak | A1 | central venous catheter |  | X |
| A12 | 10-2018 | Outbreak | A10 | anal swab | X | X |
| A13 | 10-2018 | Outbreak | A11 | pharyngeal swab | X | X |
| A14 | 10-2018 | Outbreak | A12 | anal swab | X | X |

Table S2: Average Nucleotide Identity (ANI; in %) between representative *E. cloacae* complex isolates and reference genomes. ANI > 95% (blue) indicates identity on species level; ANI > 98% (green) indicates identity on subspecies level.

| Isolate ID | SNP cluster | *E. asburiae* ATCC35953 | *E. asburiae* L1 | *E. bugandensis* isolate EB-247 | *E. bugandensis* IF3SW-P2 | *E. ludwigii* P101 | *E. ludwigii* EN-119 | *E. roggenkampii* MGH132 | *E. roggenkampii* DSM 16690 | *E. hormaechei subsp. steigerwaltii* strain DSM 16691 | *E. hormaechei subsp. steigerwaltii* strain 34998 | *E. hormaechei subsp. oharae* strain 34978 | *E. hormaechei subsp. oharae* strain DSM 16687 | *E. hormaechei subsp. hoffmannii* strain DSM 14563 | *E. hormaechei subsp. hoffmannii* strain AR 0365 |
| --- | --- | --- | --- | --- | --- | --- | --- | --- | --- | --- | --- | --- | --- | --- | --- |
| 8 | C | 88.7 | 88.6 | 89.2 | 89.2 | 87.2 | 87.2 | 88.8 | 88.6 | 98.9 | 99.1 | 97.2 | 97.8 | 95.9 | 95.9 |
| 13 | M | 91.6 | 91.6 | 98.8 | 98.8 | 88.3 | 88.3 | 91.5 | 91.5 | 89.1 | 89.1 | 89.1 | 89.2 | 88.9 | 89.0 |
| 15 | E | 88.7 | 88.6 | 89.1 | 89.1 | 87.2 | 87.2 | 88.6 | 88.6 | 99.0 | 99.0 | 97.2 | 97.9 | 96.0 | 95.9 |
| 17 | Q | 88.7 | 88.5 | 89.1 | 89.2 | 87.2 | 87.2 | 88.9 | 88.7 | 97.2 | 97.2 | 99.1 | 97.3 | 96.0 | 95.9 |
| 42 | P | 93.3 | 93.2 | 91.5 | 91.5 | 88.6 | 88.6 | 98.4 | 98.6 | 88.6 | 88.7 | 88.7 | 88.7 | 88.5 | 88.5 |
| 49 | O | 89.0 | 88.8 | 88.4 | 88.4 | 99.0 | 99.0 | 88.8 | 88.7 | 87.2 | 87.3 | 87.3 | 87.5 | 87.1 | 87.4 |
| 51 | D | 88.7 | 88.6 | 89.2 | 89.2 | 87.3 | 87.2 | 88.7 | 88.6 | 99.1 | 99.1 | 97.2 | 97.8 | 96.0 | 95.9 |
| 69 | S | 88.7 | 88.6 | 89.1 | 89.1 | 87.2 | 87.2 | 88.7 | 88.6 | 99.0 | 99.0 | 97.2 | 97.8 | 96.0 | 95.9 |
| 91 | B | 88.6 | 88.6 | 89.1 | 89.1 | 87.2 | 87.1 | 88.7 | 88.6 | 99.1 | 99.0 | 97.1 | 97.8 | 95.9 | 95.9 |
| 146 | K | 88.9 | 88.8 | 89.1 | 89.1 | 87.1 | 87.1 | 88.8 | 88.6 | 95.9 | 95.9 | 95.9 | 95.9 | 99.0 | 99.1 |
| 154 | A | 88.7 | 88.5 | 89.0 | 89.1 | 87.2 | 87.1 | 88.7 | 88.5 | 99.0 | 99.0 | 97.2 | 97.8 | 95.9 | 95.9 |
| 164 | N | 97.0 | 96.9 | 91.6 | 91.6 | 88.7 | 88.7 | 93.3 | 93.3 | 88.6 | 88.6 | 88.5 | 88.7 | 88.3 | 88.5 |
| 166 | H | 88.7 | 88.6 | 89.1 | 89.1 | 87.2 | 87.2 | 88.7 | 88.6 | 98.9 | 99.0 | 97.2 | 97.8 | 95.9 | 95.9 |
| 173 | I | 88.6 | 88.5 | 89.0 | 89.0 | 87.1 | 87.0 | 88.6 | 88.5 | 95.9 | 95.9 | 95.9 | 96.0 | 99.1 | 99.1 |
| 231 | R | 97.0 | 97.0 | 91.7 | 91.7 | 88.8 | 88.8 | 93.3 | 93.3 | 88.7 | 88.7 | 88.7 | 88.8 | 88.5 | 88.6 |
| 232 | L | 88.6 | 88.4 | 89.1 | 89.1 | 87.1 | 87.1 | 88.7 | 88.5 | 95.9 | 95.9 | 95.9 | 96.0 | 99.2 | 99.1 |
| 234 | J | 88.6 | 88.4 | 89.0 | 89.0 | 87.1 | 87.1 | 88.6 | 88.4 | 95.9 | 95.9 | 95.9 | 95.9 | 99.1 | 99.6 |
| 238 | F | 88.7 | 88.5 | 89.1 | 89.1 | 87.2 | 87.2 | 88.8 | 88.6 | 99.0 | 99.0 | 97.2 | 97.8 | 95.9 | 95.9 |
| 239 | G | 89.5 | 89.3 | 92.3 | 92.4 | 87.5 | 87.5 | 89.5 | 89.3 | 98.1 | 98.1 | 96.3 | 96.9 | 95.2 | 95.2 |

Table S3 (separate file): Pairwise FTIR spectrum similarities of 239 *E. cloacae* complex isolates. First isolates are shown in blue, additional sequenced isolates are shown in orange. Isolate spectrum pairs grouped in one FTIR cluster are marked by a box.

Table S4: Average Nucleotide Identity (ANI; in %) between outbreak *E. cloacae* complex isolates and reference genomes. ANI > 95% (blue) indicates identity on species level; ANI > 98% (green) indicates identity on subspecies level.

| Isolate ID | SNP cluster | *E. roggenkampii* MGH132 | *E. roggenkampii* DSM 16690 | *E. hormaechei subsp. steigerwaltii* strain DSM 16691 | *E. hormaechei subsp. steigerwaltii* strain 34998 | *E. hormaechei subsp. hoffmannii* strain DSM 14563 | *E. hormaechei subsp. hoffmannii* strain AR 0365 |
| --- | --- | --- | --- | --- | --- | --- | --- |
| A4 | AA | 88.7 | 88.4 | 95.9 | 95.9 | 99.1 | 99.0 |
| A12 | AD | 98.3 | 98.5 | 88.6 | 88.6 | 88.5 | 88.7 |
| A13 | AC | 88.7 | 88.5 | 95.9 | 96.0 | 99.2 | 99.6 |
| A14 | AB | 88.8 | 88.6 | 99.0 | 99.0 | 95.9 | 95.9 |

Table S5: Influence of spectrum wavenumber range used for clustering of 53 *E. cloacae* complex isolates. Adjusted Rand Index (ARI) is calculated for comparison of FTIR spectrum clustering with sequence type (ST) or SNP clustering obtained from WGS analysis. The similarity cut-off value (percentage), which provides maximum ARI, is given in brackets.

| Wavenumber range | Region | Maximum ARI | |
| --- | --- | --- | --- |
|  |  | ST | SNP cluster |
| 4,000–500 cm^-1^ | Acquired spectrum | 0.701 (89%) | 0.507 (91%) |
| 3,000–2,800 cm^-1^ | Fatty acids | 0.249 (97%) | 0.220 (97%) |
| 1,800–1,500 cm^-1^ | Proteins | 0.181 (94%) | 0.210 (93%) |
| 1,500–1,200 cm^-1^ | Mixed region | 0.562 (92%) | 0.382 (92%) |
| 1,200–900 cm^-1^ | Polysaccharides | 0.718 (73%) | 0.526 (72%) |
| 900–700 cm^-1^ | Fingerprint region | 0.651 (78%) | 0.523 (79%) |
| 1,300–800 cm^-1^ | Used by manufacturer | 0.716 (77%) | 0.527 (77%) |

Figure S1: Phylogenetic analysis of 53 *E. cloacae* complex isolates.

First isolates from patients are marked with an asterisk. SNP clusters are shown as shaded boxes. MLST and species were derived from WGS data. *E. b.*: *E. bugandensis*; *E. a.*: *E. asburiae*; *E. h. s.*: *E. hormaechei subsp. steigerwaltii*; *E. h. o.*: *E. hormaechei subsp. oharae*; *E. h. h.*: *E. hormaechei subsp. hoffmannii*; *E. l.*: *E. ludwigii*; *E. r.*: *E. roggenkampii*


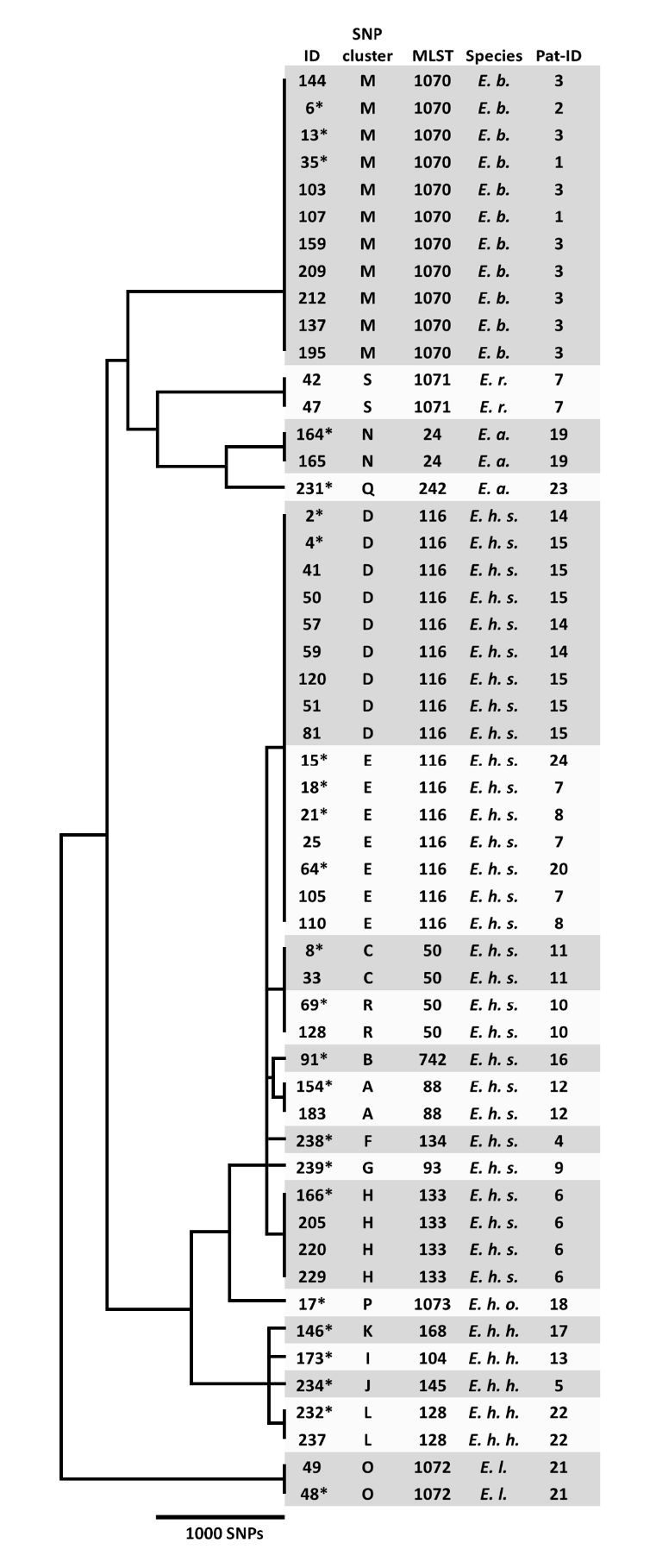


Figure S2: Phylogenetic analysis of *E. cloacae* complex outbreak isolates.

SNP clusters are shown as shaded boxes. MLST and species were derived from WGS data. *E. h. s.*: *E. hormaechei subsp. steigerwaltii*; *E. h. h.*: *E. hormaechei subsp. hoffmannii*; *E. r.*: *E. roggenkampii*


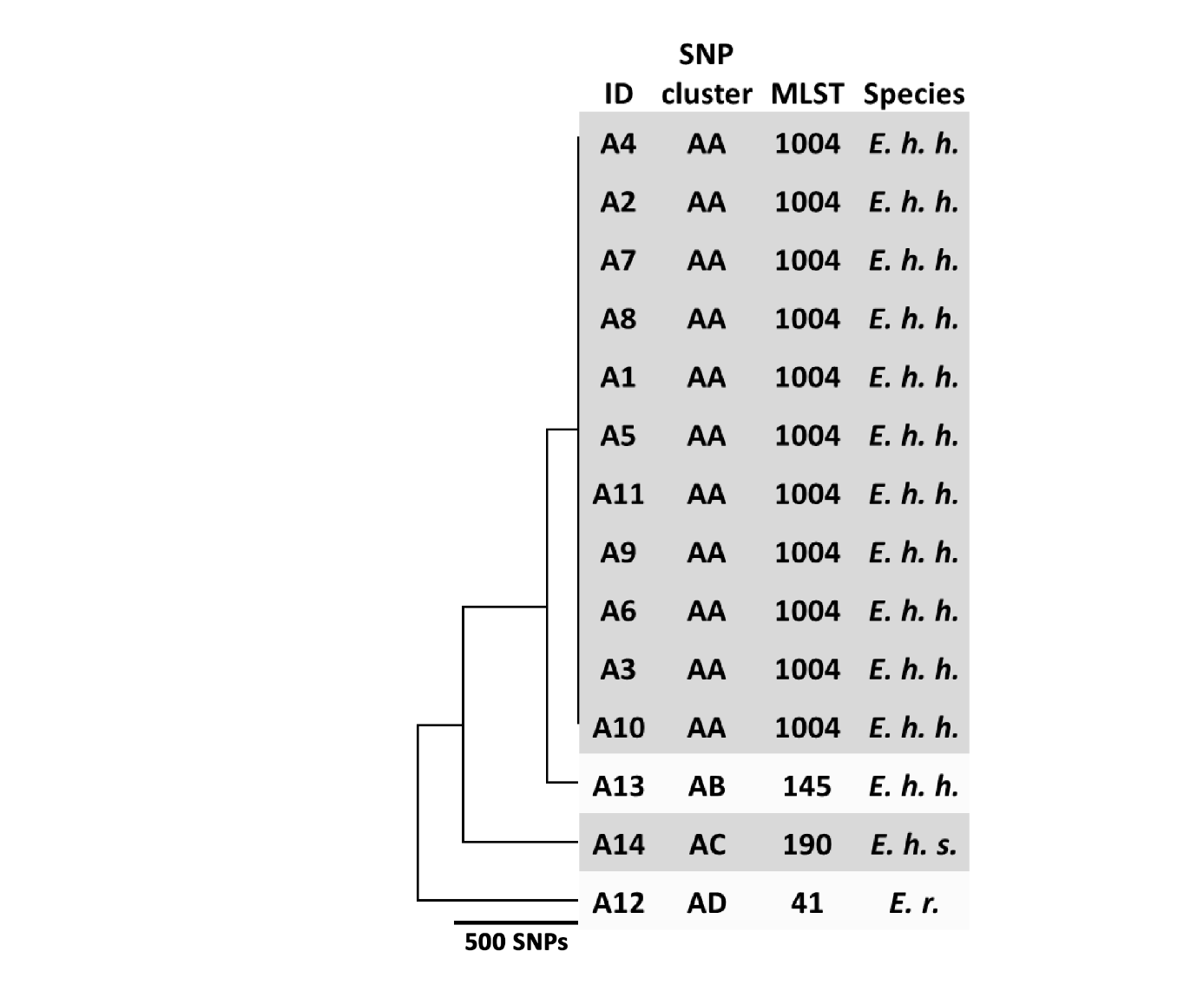

Supplement: Supplementary file 1 [file Data_Sheet_1.docx]
